# Differences in Dietary Intake Exist among U.S. Adults by Diabetic Status Using NHANES 2009–2016

**DOI:** 10.3390/nu14163284

**Published:** 2022-08-11

**Authors:** Luotao Lin, Fengqing Zhu, Edward J. Delp, Heather A. Eicher-Miller

**Affiliations:** 1 Department of Nutrition Science, College of Health and Human Sciences, Purdue University, West Lafayette, IN 47907, USA; 2 School of Electrical and Computer Engineering, College of Engineering, Purdue University, West Lafayette, IN 47907, USA

**Keywords:** food frequency, energy contribution, food category, diet choice, diabetes, insulin

## Abstract

The objective was to determine the most frequently consumed food items, food subcategories, and food categories, and those that contributed most to total energy intake for the group of U.S. adults reporting taking insulin, those with type 2 diabetes (T2D) not taking insulin, and those without diabetes. Laboratory tests and questionnaires of the National Health and Nutrition Examination Survey 2009–2016 classified 774 participants reporting taking insulin, 2758 participants reporting T2D not taking insulin, and 17,796 participants without diabetes. Raw and weighted frequency and energy contributions of each food item, food subcategory, and food category were calculated and ranked. Comparisons among groups by broad food category used the Rao–Scott modified chi-square test. Soft drinks ranked as the 8th and 6th most consumed food subcategory of participants with T2D not taking insulin and those without diabetes, and contributed 5th and 2nd most to energy, respectively. The group reporting taking insulin is likely to consume more protein foods and less soft drink compared to the other two groups. Lists of the most frequently reported foods and foods contributing most to energy may be helpful for nutrition education, prescribing diets, and digital-based dietary assessment for the group reporting taking insulin.

## 1. Introduction

Diabetes is a chronic disease highly influenced by diet where individuals either do not produce enough insulin to regulate blood glucose level or they are not able to use the insulin they produce to effectively move blood glucose into body cells [1]. Over time, diabetes can cause additional serious health problems, including heart disease, vision loss, and kidney disease. About 34.2 million U.S. individuals (10.5% of the U.S. population) had diabetes—type 1 (T1D), type 2 (T2D), and gestational diabetes—in 2020 [2].

Both those with T1D and T2D may use injectable insulin to control their blood glucose levels. Proper insulin dosing to control blood glucose levels and reduce the risk of severe consequences is dependent on dietary intake, particularly the frequency and energy from macronutrients: carbohydrate, protein, and fat [3,4]. Thus, quantification of the foods comprising dietary intake may be used to inform insulin dosing algorithms to enhance blood glucose level control [5,6], and knowledge of the foods comprising overall dietary intake may also aid in the creation of meal plans, inform substitutions, and other interventions. Furthermore, it is likely that insulin use may differentiate food and beverage choice and intake among those reporting diabetes, as dietary attention to manage blood glucose levels is necessary for insulin treatment. Glycemic control through insulin administration, as well as through oral antidiabetic agents, may be influenced by the macronutrients in the foods and also through insulin resistance, which may be boosted by some foods [7]. Therefore, among those with diabetes, whether T1D or T2D, using insulin may homogenize dietary behaviors compared to those with T2D but not taking insulin, and both groups may vary from the group of adults who do not have diabetes. Yet, very little information on the food intake of those taking insulin or those with T2D but not taking insulin is available [8]. Previous studies found that groups with diabetes had low diet quality or diets with little alignment to dietary recommendations [9,10] or evidence of significant differences in nutrient intake between groups with and without diabetes. Specifically, the group with diabetes consumed more calcium and sodium than the group without diabetes, and the group with continuous glucose monitoring and/or continuous subcutaneous insulin infusion consumed more protein and lipids but less carbohydrates and fiber than the group without continuous glucose monitoring and/or continuous subcutaneous insulin infusion [9,10,11]. Further studies evaluating differences among those using and not using insulin were not identified.

Therefore, the objectives of this study were to (1) determine and compare the lists of the most frequently consumed food items, food subcategories, and food categories and the food items, food subcategories, and food categories that contributed most to energy intake, and (2) compare the differences in the frequency of reported intake of the broad food categories and the contributions to energy of the broad food categories among those reporting taking insulin, those with T2D but not taking insulin, and those without diabetes among U.S. adults aged 18 years old or older using data from the National Health and Nutrition Examination Survey (NHANES) 2009–2016.

## 2. Materials and Methods

### 2.1. Participants and Dataset

NHANES is a cross-sectional survey that is conducted by the National Center for Health Statistics (NCHS) of the U.S. Centers for Disease Control and Prevention to assess the health and nutritional status of the non-institutionalized civilian population in the U.S. [12]. Participants’ sociodemographic information was obtained via questionnaires during the in-person household interview, which included age, sex, race/ethnicity, and poverty to income ratio (PIR). Participants’ BMI was obtained from health examination. The NCHS Research Ethics Review Board approved the survey, and all participants consented to participate [13].

### 2.2. Analytic Sample

NHANES 2009–2016 was used to include the most recent available dietary data and sufficient sample size. Reliable first 24-hour dietary recall used in the analysis, reliable laboratory test results, and valid answers to diabetic-related questions were drawn from adults aged 18 or older to make up the analytic sample data.

**Figure 1:**
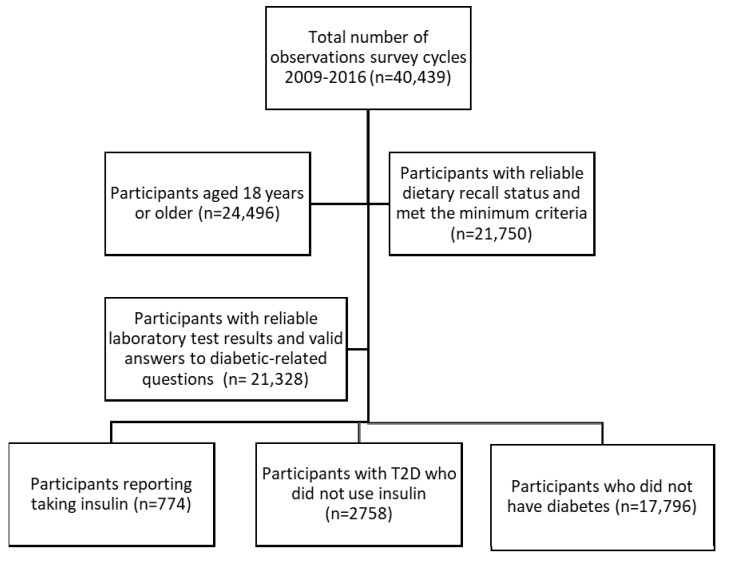
Flowchart representing sample-size attrition and reasons for inclusion.

NHANES diabetes-related questions and laboratory test results were used to divide the sample into three groups: participants reporting taking insulin, participants with diabetes not taking insulin, and participants without diabetes. The number of the group reporting taking insulin was determined by the question “Are you/is the sample person now taking insulin?” Those participants with diabetes who were not taking insulin were identified by subtracting those using insulin from the total number of participants with diabetes. Diabetes was classified by self-reporting, being told they had diabetes by a doctor or taking medications that lowered glucose, or by fasting plasma glucose concentration ≥126 mg/dL or hemoglobin A1c ≥ 6.5% [14]. Since T1D was classified when a participant reported being diagnosed with diabetes before the age of 30 years and reporting continuous insulin use since diagnosis [15], the group reporting taking insulin encompassed all those with T1D, so participants with diabetes not taking insulin were in the same group as participants with T2D but not taking insulin. Those participants without diabetes were identified by subtracting the number of participants with diabetes from the sample. As a result, the study sample of foods and beverages used in the analysis was drawn from 774 participants reporting taking insulin, 2758 participants with T2D but not taking insulin, and 17,796 participants who did not have diabetes (Figure 1).

### 2.3. Sociodemographic and Sample Characteristics

Data used to describe the characteristics of those from which dietary information was included in the analysis were survey year (2009–2010, 2011–2012, 2013–2014, and 2015–2016) and self-reported sex (male or female), race/ethnicity (grouped as Hispanic, non-Hispanic white, non-Hispanic black, and other, including multirace), age (grouped as 18–34, 35–49, and 50–80 years), and PIR, which was the reported household income divided by the federal poverty guideline for household income, and grouped as 0–0.99 (below poverty threshold), 1–1.99, 2–2.99, 3–3.99, and >4 based on the U.S. Census Bureau’s definition of being in poverty and maintaining an equitable PIR range for low-income categories [16]. BMI was classified as underweight and normal weight (<25 kg/m^2^) because only a few participants were underweight, overweight (25.0–29.9 kg/m^2^), and obese (≥30.0 kg/m^2^) [17].

### 2.4. Dietary Assessment

The U.S. Department of Agriculture (USDA) Automated Multiple-Pass Method [18] was used to collect dietary recall recorded during the physical health examination, where participants reported all of the foods consumed during the previous 24-hour period, including information on the time of intake, amount and type of each food, and detailed food descriptions [19]. Each reported food item was then linked to the USDA’s Food and Nutrient Database for Dietary Studies (FNDDS) [20,21,22,23] to assign 8-digit food codes. The USDA food codes were also used to sort the reported foods into the What We Eat in America (WWEIA) food categories and subcategories [24].

### 2.5. Statistical Analysis

The Rao–Scott modified chi-square test was used to compare sociodemographic variables created from the survey data among the three groups.

All analysis was based on the foods and beverages reported as consumed within the three groups and not the individual participant. The weighted frequencies of reported foods were computed by determining the weighted sum of the number of food items, food subcategories, and food categories using the population ratio approach:

$$\overset{i=1}{\underset{n}{\sum }}\left(R_{i}W_{i}\right)$$

where *n* = the sample size, *i* = each participant, *R_i_* = number of reports of individual food code for the *i*th participant, and *W_i_* = dietary day 1 sample weight for the *i*th participant [25,26]. The number of reports of foods for each participant was multiplied by the dietary day 1 sample weight, and then summed to yield the total weighted frequency of the particular food or beverage for each respective group, which represented how often that food or beverage was consumed by the respective group in the U.S. in 1 day. The weighted frequency of the food or beverage was also divided by the total weighted frequency of all foods or beverages reported in a single day to get the weighted percentage of the frequency of that food or beverage being consumed out of all reported foods and beverages consumed.

The weighted energy contribution of reported food items, food subcategories, and food categories was computed by determining the weighted sum using the population ratio approach:

$$\overset{i=1}{\underset{n}{\sum }}\left(E_{i}W_{i}\right)$$

where *n* = the sample size, *i* = each participant, *E_i_* = total energy for an individual food code for the *i*th participant, and *W_i_* = dietary day 1 sample weight for the *i*th participant [25,26]. The energy contributed by a particular food or beverage was first summed for each participant and then multiplied by the dietary day 1 sample weight of the participant, then summed for all participants in the sample for each group, which represented how much energy that food or beverage contributed to the total energy for the respective group in the U.S. in 1 day. The weighted energy of the particular food or beverage was also divided by the total weighted energy from all reported foods and beverages to get the weighted percentage of energy from that food or beverage out of the energy estimated from all reported foods and beverages.

Kruskal–Wallis tests were used to compare the differences among the top 10 ranking lists with the various food hierarchy levels in the three groups, while Rao–Scott modified chi-square goodness-of-fit tests were used to compare the frequency and energy contributions of the individual food categories at the broad food category level among the three groups. Significant differences were indicated when *p* < 0.05/42 or *p* < 0.0012 using a Bonferroni-type adjustment for multiple comparisons for food intake among the 14 broad food categories × 3 groups. Survey weights, or the reciprocal of sample inclusion probability, were used in all computations to allow for inference to the non-institutionalized U.S. population. Further adjustments were performed to account for the clustering and stratification inherent to the survey design.

All analyses were completed in SAS 9.4 (SAS Institute Inc., Cary, NC, USA).

## 3. Results

### 3.1. Sociodemographic and Sample Characteristics

The sociodemographic and sample characteristics of participants taking insulin, with T2D but not taking insulin, and participants without diabetes are shown in Table 1. Sex, age, race/ethnicity, PIR, and BMI were significantly different among the three groups.

Most participants reporting taking insulin, and those with T2D but not using insulin were aged 50–80 years. Participants without diabetes were more evenly distributed in the three age-groups (18–34, 35–49, 50–80). About half of the group using insulin and with T2D but not taking insulin had PIR < 2. Furthermore, 28.1% of the group reporting taking insulin had PIR below the poverty threshold (PIR < 1). The group without diabetes had higher incomes, with more than 25% of participants having a PIR greater than 4. In addition, BMI ≥ 30 was heavily represented among participants in the group reporting taking insulin (64.7%) and in the group reporting T2D but not taking insulin (58.1%). The three categories of BMI were evenly distributed in the group without diabetes (33.9%, 33.0%, and 33.1%, respectively).

### 3.2. The Most Frequently Consumed Food Items, Food Subcategories, and Food Categories

The top 10 most frequently reported food items, food subcategories, and food categories for the group reporting taking insulin, those with T2D but not using insulin, and participants without diabetes are shown in Table 2, Table 3 and Table 4.

The ranked lists were not statistically different (*p*-value = 0.69, 0.73, 0.96, respectively), however they allow insight to the dietary context and patterns of these 3 groups. Foods are presented in descending weighted frequency order by their FNDDS short food descriptions. In these frequency lists, 7 food items (Table 2), 6 food subcategories (Table 3), and 8 food categories (Table 4) are common across the three groups, but their rankings were varied (but not in a statistically significant manner). Notably, diet soft drink was the 4th most frequently consumed food subcategory among the group reporting taking insulin, the 7th among those with T2D but not taking insulin, and did not appear in the top 10 food subcategory frequency list among those without diabetes (Table 3). Regular soft drink did not appear in the top 10 food subcategory frequency list for the group reporting taking insulin, but was the 8th most frequently consumed food subcategory among those with T2D but not taking insulin, and the 6th most frequently consumed food subcategory among participants without diabetes (Table 3). In addition, sugar substitutes and cold cuts and cured meats ranked 8th and 10th, respectively, in the top 10 list of the group reporting taking insulin but not the other groups (Table 3), while other vegetables and combinations ranked in top 10 list of the group reporting T2D but not taking insulin (9th) and in the group without diabetes (8th) but not for those taking insulin (Table 3).

### 3.3. The Highest Energy Contributing Food Items, Food Subcategories, and Food Categories

The top 10 highest energy contributing food items, food subcategories, and food categories for the participants reporting taking insulin, those with T2D but not using insulin, and participants without diabetes are shown in Table 5, Table 6 and Table 7.

Similar to the frequency lists, the list of top energy contributing foods and beverages were not statistically different (*p*-value = 0.11, 0.34, 0.61, respectively), but provide context to the diets consumed by the 3 groups. Foods are presented in descending weighted order according to their energy contribution by their FNDDS short food descriptions. In these energy contribution lists, 5 food items (Table 5), 6 food subcategories (Table 6), and 5 food categories (Table 7) were similar among the three groups, but their rankings varied (but not statistically significant manner). Regular soft drinks did not rank in the top 10 most energy contributing food subcategories list among the group reporting taking insulin, but was ranked 5th among those with T2D but not taking insulin, and 2nd among participants without diabetes (Table 6). Furthermore, the group reporting taking insulin consumed less energy from food subcategories with added sugars compared with the other two groups (cakes and pies were 6th and cookies and brownies 7th among the group reporting taking insulin; cakes and pies were 3rd and cookies and brownies 6th among those with T2D but not taking insulin; cookies and brownies were 8th and cakes and pies 9th among participants without diabetes). More protein food subcategories ranked high within the top 10 list (whole pieces of chicken ranked 3rd place, eggs and omelets ranked 4th place, meat mixed dishes ranked 5th place, cold cuts and cured meats ranked 10th) among the group reporting taking insulin compared to the other two groups (whole pieces of chicken ranked 4th place, eggs and omelets 9th place, meat mixed dishes 10th place in those with T2D but not using insulin, and whole pieces of chicken ranked 5th place in participants without diabetes) (Table 6). In addition, whole wheat bread and white potato ranked 6th and 8th (Table 5) in the top 10 lists of the group reporting taking insulin, but was not ranked in the lists of the other groups, while French fries ranked 5th in the top 10 lists of the group reporting T2D but not taking insulin and 3rd in the group without diabetes, but was not ranked in the lists of the group taking insulin (Table 5). Moreover, lite beer and beer ranked in the top 10 lists of the group without diabetes (5th in Table 5 and 4th in Table 6) but was not ranked in the lists of the other groups. Fruit-flavored soft drink ranked 10th only in the top 10 lists of the group without diabetes (Table 5).

### 3.4. Broad Food Category Intake by Frequency and Energy Contribution

The results of the comparisons of the frequencies and energy contributions of the individual broad WWEIA food or beverage categories among the three groups are shown in Table 8.

The percentage of intake from the broad categories of grains and alcohol were statistically significantly different in both intake frequency and energy contribution across the three groups. From the group reporting taking insulin to those with T2D but not using insulin to participants without diabetes, grains exhibited a decreasing trend in consumption frequency and energy contribution, while alcohol showed an increasing trend. The percentage of snacks/sweets were statistically significantly different in consumption frequency, but not energy contribution among the three groups, which showed an increasing trend from the group reporting taking insulin to those with T2D not using insulin to participants without diabetes. The percentage of intake from protein foods, vegetables, beverages, water, and other were statistically significantly different in energy contribution across the three groups. From the group reporting taking insulin to participants without diabetes, protein foods and vegetables exhibited a decreasing trend in percentage of energy contribution, while beverages, water, and other showed an increasing trend.

## 4. Discussion

Few studies have investigated the diets of those reporting taking insulin [9,10,11], nor have these studies included consideration of the dietary intake contributions of foods to both energy and frequency of intake. To our knowledge, this is the first study to investigate and compare the diets of the group reporting taking insulin, those with T2D but not taking insulin, and participants without diabetes. Participants with varying diabetes and insulin-use status showed significant differences in both the frequency of which certain broad food or beverage categories are consumed and the energy contributions attributed to certain food or beverage food categories, implying that insulin-use status may be linked to dietary behaviors and the importance of certain foods or beverages in the overall diet. In some of the broad WWEIA food categories, the statistically significant differences observed in the frequency were also observed in energy contribution. Both the percentage of consumption frequency and energy contribution of grains (wheat bread) and alcoholic beverages (beer) were statistically significantly different among the three groups.

The results also included statistically significant findings where either the frequency or energy contribution percentage differed among the groups but not both. Beverages, water, and the “other” (nutrition powder) broad food categories did not exhibit significantly different percentage of frequency of intake across the three groups, but the share of energy contributed by these broad food categories was lower among the participants reporting taking insulin compared to those without diabetes. The share of energy contributed by protein (eggs and omelets, cold cuts and cured meats) and vegetables (tomatoes and lettuce) was also greater among the group reporting taking insulin compared with participants without diabetes. Snacks/sweets (cookies and brownies, and cakes and pies) exhibited a lower percentage of consumption frequency among the group reporting taking insulin compared with participants without diabetes, but the percentage of energy intake attributed to snacks/sweets was not significantly different across all three groups.

Results of this study suggest the group reporting taking insulin exhibited a pattern of higher quality regarding dietary intake for the foods they consumed frequently and that contributed most to energy compared to those with T2D not using insulin and participants without diabetes. However, actual diet quality analyses among the three groups at the person rather than food unit of analysis level needs further evaluation. Since older adults accounted for the majority of the group reporting taking insulin, this finding is consistent with previous studies showing that older adults have higher-quality diets [11,25,27,28]. Yet, almost two thirds of the group that reported taking insulin and more than half of the group with T2D but not taking insulin were obese, which has previously been inversely related to diet quality [29,30] as has poverty-income ratio [31]. The proportion of various race/ethnicities among the groups and gender distribution were also differentially distributed and may have influenced the dietary quality, selection, and patterns of foods among the three groups in the study. Other factors may have also influenced the patterns observed, such as the length of time since diagnosis or since insulin use [32,33,34]. Yet, this study focused on the foods themselves, and not the person as the unit of analysis. Therefore, the personal characteristics of the individuals in each of the three groups compared were not controlled for in the analysis.

In addition, the finding that the group reporting taking insulin may have a higher diet quality is also supported by a previous study [34], suggesting that those with diabetes who are using insulin engage in dietary behaviors promoting a more healthful mix of dietary intake compared with those who do not have diabetes and those with T2D but not taking insulin. The group using insulin reported a higher percentage of consumption frequency of grains, energy contribution from grains, vegetables, and protein foods; and lower percentage consumption frequency of alcohol and sweets/snacks and energy contributed by beverages and alcohol compared to participants without diabetes. One explanation is that grains, protein foods, and vegetables have been promoted as foods to consume to help manage blood glucose control for those taking insulin. Carbohydrate intake is highly related to postprandial glycemia, whole grains are a source of carbohydrates that have a favorable influence on postprandial blood glucose levels [35]. Protein foods may also aid dietary compliance and weight loss maintenance, which is beneficial for diabetes [36,37]. Vegetable consumption has been associated with the improvement of diabetes due to benefits offered by the bioactive compounds comprising them including dietary fiber, resistant starch, antioxidant vitamins, phytochemicals (polyphenols, tannins, and others) and minerals [38,39,40], which may explain why the group reporting taking insulin selected a higher percentage of their calories from vegetables compared with those who do not have diabetes. Furthermore, alcohol, sweets/snacks, and beverages (heavily influenced by sweetened beverages) are foods that those taking insulin may try to avoid due to their negative impact on glycemic control. These foods that may add solid fats or added sugar intake and should be reduced according to the Dietary Guidelines for Americans, 2020–2025 [41,42].

With regard to differences among the percentage of beverage and alcohol intake of the groups, previous studies found that those with diabetes drink less alcohol than those without diabetes [43,44], which supports the findings here that those who used insulin and those with T2D not using insulin, consumed lower percentages of alcohol than participants without diabetes. Even though the long-term effect of alcohol consumption on glycemic management remains to be investigated [45], heavy alcohol consumption is associated with an increased incidence of diabetes [45] and alcohol consumption may be a negative indicator of diabetes self-care behavior [46]. The beverage intake of the group using insulin further varied from the other two groups in the pattern of energy contribution from soft drinks (Table 5), which was supported by the statically significant lower percentage of energy contribution of beverages at the broad food category level in the group reporting taking insulin compared to the group without diabetes (Table 8). Yet, the percentage of the frequency of consuming beverages at the broad food category level was not significantly different among these two groups (Table 8) and those reporting taking insulin consumed diet soft drinks more frequently (Table 3), which may be viewed as an alternative choice that avoids added sugars. Interestingly, this significant difference in energy from beverages was not observed in the categories of “cookies and brownies” or “cakes and pies” (Table 8), which are categories that also have high amounts of added sugars but may not have similar alternatives. Furthermore, within the same food category, the group taking insulin may prefer to choose white potato rather than the fried version of French fries that the group with T2D not using insulin and the group without diabetes choose (Table 5).The findings related to lower frequency of sweets/snacks and beverages among those using insulin compared to those without diabetes is supported by a previous study where participants with diabetes consumed lower amounts of sweets and beverage (juice) compared to those without diabetes [44]. Sweets/snacks and beverages, especially sweetened beverages that heavily add to energy intake, are typically high in added sugars and may to lead nonalcoholic fatty liver disease [47]. Excessive added sugar intake is not only a significant contributor to weight gain that may lead to increased risk of diabetes but also increased dietary glycemic load and fructose metabolism that may lead to inflammation and insulin resistance [48]. Fructose consumption, for instance, has been associated to diabetes onset and progression as well as nonalcoholic fatty liver disease development and progression, stressing the role of metabolic syndrome with increase deposition of epicardial fat and worsening clinical outcomes [49,50]. In addition, it should be noted that the literature reviewed here includes studies focused on those with diabetes, not specifically those using insulin, due to the extremely limited evidence of dietary intake among those using insulin [39,40,44]. Therefore, the findings here should be further explored among those taking insulin.

Limitations of this analysis may be due to the aggregation of foods to subcategories and categories that may obscure specific food items or types of food that could be related to many of the characteristic differences among groups and that is agnostic of the individual when statistical comparisons were made. As mentioned, the focus of the analysis was on the foods, not the individual person, meaning sociodemographic characteristics were not adjusted for in the analysis. The addition or removal of specific foods or beverages from a food category has the potential to change statistically significant comparisons, so the results and interpretation should be viewed as one perspective on the dietary intake among these three groups, keeping in mind that analysis of alternative food categorization, splitting to subcategories, or itemization may yield differential results. However, the application of the WWEIA food categories lends standardization and consistency to other reporting by USDA and other researchers [51,52,53]. In addition, the ranking lists (Table 2, Table 3, Table 4, Table 5, Table 6 and Table 7) were generated from the foods and beverages reported over all times of the day, so separating out the most frequently consumed foods and foods that highly contributed to energy intake for different times or meals of the day may be helpful to inform interventions or dosing algorithms as the timing of dietary intake and combinations of foods consumed at the same time influences glucose metabolism [54,55,56,57,58]. It is important to note that the comparison of ranking lists among the 3 groups did not consider the amount or magnitude of the consumption frequency or energy contribution of different food hierarchy levels, it only considered sequence of ranking order. Furthermore, the comparisons of these ranked lists were limited to the top 10 foods which were not different between groups. The inclusion of more foods in the ranked lists may have yielded different results. Yet, the lists contribute a descriptive context of the diets of these three groups that is not available elsewhere in the literature and may be helpful to inform menus, aid the development of nutrition assessment tools, and create nutrition interventions. Furthermore, the NHANES dietary data this study used are only a 1-day dietary recall of the participants, which may not be accurate due to participants’ memory and may not represent the usual dietary intake [59]. Moreover, misreported dietary intake may have been presented in this study sample resulting in errors in energy, frequency, food item, subcategory, and category representation and comparisons [60]. Those with diabetes and those reporting taking insulin may exhibit different patterns of misreporting compared with other population groups [44]. Previous studies have shown that dietary intake misreporting and especially underreporting is more prevalent in overweight and obese individuals compared to normal weight individuals [61,62,63], and prevalence of underreporting in diabetic patients may be even higher [64], considering that obesity is a risk factor of diabetes that 81% of diabetic patients are obese [65]. Another previous study pointed out that energy misreporters are prone to misreport foods that highly contributed to glycemic index and glycemic load such as underreporting foods including sugars, cookies, and milk products and overreporting foods including fruit and vegetables [63]. In the future, studies evaluating the differences in misreporting by diabetic status should be evaluated. Furthermore, the dietary behaviors of families may be related and the habits of children may predict future risk of metabolic syndrome. Therefore, the relationship of the dietary intake of parents using insulin and their children should be evaluated.

There are several implications and applications for the results of this study. Diabetes is a chronic disease that is highly related to diet [66]. The lists of foods generated in this study may be used to fill the gap in knowledge of the diets for the group reporting taking insulin or diabetic patients among U.S. adults [9,11], specifically, menus and dietary interventions developed to improve diabetes management may be informed by the lists. Although frequently consumed foods and those contributing most to energy may not satisfy the preferences of every person, they are generally accepted as indicated by their selection in the population. Those using insulin may be more likely to adhere to a diet they accept, thus forming healthier eating habits. Second, the findings of this study may inform future studies to gain a greater knowledge of the diets of those using insulin. For example, the list of frequently consumed foods and those most contributing to energy may inform the prioritization of foods and beverages to include in food frequency questionnaires. Third, the findings of this study may aid the development of nutrition assessment tools as researchers should concentrate efforts on developing the most accurate identification methods for the foods and beverages that are consumed the most frequently and contribute the most to total energy intake in the group they are tailored for [25].

## 5. Conclusions

Differences in dietary intake exist among U.S. adults by insulin use and diabetes status using NHANES 2009–2016. The group reporting taking insulin consumed grains more frequently, while consuming snack/sweet and alcohol less frequently compared to participants without diabetes. Protein, grains, and vegetables contributed more to the group reporting taking insulin’s daily total energy intake compared to participants without diabetes, whereas beverage and alcohol contributed less. Frequently consumed foods and those contributing to energy may inform diabetes control and management; be used to tailor food recipes; and applied in consumer education, questionnaire design, and dietary assessment.

## Figures and Tables

**Table 1:** Characteristics of participants and data of U.S. adults 18 year or older taking insulin, with type 2 diabetes but not taking insulin, and without diabetes as drawn from the NHANES, 2009–2016 ^1^.

	The Group Reporting Taking Insulin (*n* = 774)	The Group Reporting T2D w/o Insulin (*n* = 2758)	The Group w/o Diabetes (*n* = 17,796)	*p*-Value ^2^
Sex				0.0003 *
Male	403 (52.1%)	1416 (51.3%)	8578 (48.2%)	
Female	371 (47.9%)	1342 (48.7%)	9218 (51.8%)	
Age in Years				<0.0001 *
18–34	44 (5.6%)	121 (4.4%)	6247 (35.1%)	
35–49	91 (11.8%)	470 (17.0%)	4633 (26.0%)	
50–80	639 (82.6%)	2167 (78.6%)	6916 (38.9%)	
Survey year				0.4
2009–2010	193 (24.9%)	766 (27.8%)	5005 (28.1%)	
2011–2012	181 (23.4%)	647 (23.5%)	4168 (23.4%)	
2013–2014	181 (23.4%)	660 (23.9%)	4387 (24.7%)	
2015–2016	219 (28.3%)	685 (24.8%)	4236 (23.8%)	
Race/Ethnicity				<0.0001 *
Hispanic	205 (27.7%)	859 (31.1%)	4448 (25.0%)	
White	276 (35.7%)	905 (32.8%)	7517 (42.2%)	
Black	229 (29.6%)	692 (25.1%)	3640 (20.5%)	
Other	54 (7.0%)	302 (11.0%)	2191 (12.3%)	
Poverty Income Ratio				<0.0001 *
0.00–0.99	201 (28.1%)	623 (25.0%)	3776 (23.2%)	
1.00–1.99	220 (30.7%)	748 (30.0%)	4219 (25.9%)	
2.00–2.99	106 (14.8%)	384 (15.4%)	2353 (14.4%)	
3.00–3.99	66 (9.2%)	277 (11.1%)	1793 (11.0%)	
4.00–5.00	123 (17.2%)	463 (18.5%)	4156 (25.5%)	
Body Mass Index				<0.0001 *
<25	81 (10.9%)	364 (13.3%)	5981 (33.9%)	
25–29.9	182 (24.5%)	780 (28.6%)	5813 (33.0%)	
≥30	481 (64.7%)	1586 (58.1%)	5843 (33.1%)	

^1^ Total numbers do not always add to sample size due to missing values. Percentages do not always add to 100 due to rounding, ^2^ Rao–Scott modified chi-square *p*-value is shown. Analyses were adjusted for survey design, including stratification and clustering. Sample weights were appropriately constructed and applied to this analysis as directed by NCHS. Weights were rescaled so that the sum to the weights matched the survey population at the midpoint of the 8 years 2009–2016. Statistical significance for differences among three groups when *p* < 0.05 is indicated by *.

**Table 2:** Top 10 most frequently consumed foods items or beverages ^1^, unweighted and weighted frequency of reported foods items or beverages, percentage of reported foods or beverages, and standard error of percentage of reported foods or beverages among all reported foods or beverages for U.S. adults 18 year or older taking insulin, with type 2 diabetes but not taking insulin, and without diabetes drawn from 2009–2016 NHANES data ^2^.

	The Group Reporting Taking Insulin (*n* = 774)				The Group Reporting T2D w/o Insulin (*n* = 2758)				The Group w/o Diabetes (*n* = 17,796)			
Rank	What We Eat in America item ^1^	Weighted Frequency ^3,4^	Frequency ^5^	Wtd% ^4,6^ (SE)	What We Eat in America item ^1^	Weighted Frequency ^3,4^	Frequency ^5^	Wtd% ^4,6^ (SE)	What We Eat in America item ^1^	Weighted Frequency ^3,4^	Frequency ^5^	Wtd% ^4,6^ (SE)
Total		98,959,094	12,095			376,469,886	43,421			3,245,557,852	277,244	
1	Tap water	5,294,037	579	5.3 (0.4)	Tap water	20,867,328	2231	5.5 (0.2)	Tap water	202,005,485	15,400	6.2 (0.2)
2	Unsweetened bottled water	4,341,351	600	4.4 (0.3)	Unsweetened bottled water	15,815,995	2157	4.2 (0.3)	Unsweetened bottled water	114,654,421	11,820	3.5 (0.1)
3	Coffee	2,867,410	336	2.9 (0.2)	Coffee	10,982,634	1212	2.9 (0.1)	Coffee	92,688,711	6906	2.9 (0.1)
4	Tomatoes	1,583,968	179	1.6 (0.2)	Lettuce	5,520,635	597	1.5 (0.1)	White sugar	48,766,824	5271	1.5 (0.1)
5	2% reduced fat milk	1,483,662	187	1.5 (0.2)	Tomatoes	5,499,873	598	1.5 (0.1)	Lettuce	45,653,422	3684	1.4 (0.0)
6	Soft drink (cola-type, sugar-free)	1,367,532	155	1.4 (0.2)	2% reduced fat milk	5,377,691	613	1.4 (0.1)	Soft drink (cola-type)	43,801,567	4172	1.3 (0.1)
7	Lettuce	1,277,944	156	1.3 (0.1)	Soft drink (cola-type, sugar-free)	4,446,739	344	1.2 (0.1)	Tomatoes	43,656,646	3468	1.3 (0.0)
8	Soft drink (cola-type, decaffeinated)	867,827	86	0.9 (0.2)	White sugar	4,285,836	633	1.1 (0.1)	2% Fat-reduced milk	37,409,699	3298	1.2 (0.0)
9	Sugar substitute (saccharin-based)	850,553	124	0.9 (0.1)	Banana	3,928,098	530	1.0 (0.1)	Banana	31,398,643	2737	1.0 (0.0)
10	Banana	818,469	125	0.8 (0.1)	Soft drink (cola-type)	3,726,509	460	1.0 (0.1)	Whole milk	23,685,000	2338	0.7 (0.0)

^1^ 6772 food or beverage items were reported by participants from NHANES 2009–2016; ^2^ The ranked list of the top 10 most frequently reported food items for the group reporting taking insulin, those with T2D but not using insulin, and participants without diabetes were not statistically different (*p*-value = 0.69 as indicated by the Kruskal–Wallis test); ^3^ The sum of the estimated weighted frequencies for all type foods or beverages reported in 1 day among adult participants ≥18 years of NHANES 2009–2016; ^4^ Survey weights and adjustments for the complex survey design were applied to represent the non-institutionalized U.S. population; ^5^ The frequency that a food or beverage was reported without sample weights; ^6^ Derived from the weighted frequency of the individual food or beverage divided by the total weighted frequency of all foods and beverages (*n*) reported by whole sample in a single day, where *n* = 98,959,094 for the group reporting taking insulin, *n* = 376,469,886 for the group reporting T2D w/o taking insulin, and *n* = 3,245,557,852 for the group w/o diabetes. Estimated weighted percentage has been abbreviated to “Wtd %”; SE stands for standard error.

**Table 3:** Top 10 most frequently consumed What We Eat in America foods or beverage subcategories ^1^, unweighted and weighted frequency of reported foods or beverage subcategories, percentage of reported foods or beverage subcategory out of the total, and standard error of the percentage of reported foods or beverage represented by the subcategory among all reported foods or beverage subcategories for U.S. adults 18 years or older taking insulin, with type 2 diabetes but not taking insulin, and without diabetes drawn from 2009–2016 NHANES dat ^2^.

	The Group Reporting Taking Insulin (*n* = 774)				The Group Reporting T2D w/o Insulin (*n* = 2758)				The Group w/o Diabetes (*n* = 17,796)			
Rank	What We Eat in America subcategory ^1^	Weighted Frequency ^3,4^	Frequency ^5^	Wtd% ^4,6^ (SE)	What We Eat in America subcategory ^1^	Weighted Frequency ^3,4^	Frequency ^5^	Wtd% ^4,6^ (SE)	What We Eat in America subcategory ^1^	Weighted Frequency ^3,4^	Frequency ^5^	Wtd% ^4,6^ (SE)
Total		98,959,094	12,095			376,469,886	43,421			3,245,557,852	277,244	
1	Tap water	5,356,421	585	5.4 (0.4)	Tap water	21,157,722	2272	5.6 (0.2)	Tap water	205,305,226	15,673	6.3 (0.2)
2	Coffee	4,409,221	560	4.5 (0.3)	Coffee	17,061,047	2097	4.5 (0.1)	Coffee	136,685,004	11,227	4.2 (0.1)
3	Bottled water	4,341,351	600	4.4 (0.3)	Bottled water	15,815,995	2157	4.2 (0.3)	Bottled water	114,654,421	11,820	3.5 (0.1)
4	Diet soft drinks	3,718,166	391	3.8 (0.4)	Yeast breads	13,091,450	1529	3.5 (0.1)	Cheese	95,913,887	7342	3.0 (0.1)
5	Yeast bread	3,652,103	449	3.7 (0.2)	Cheese	9,577,396	969	2.5 (0.1)	Yeast breads	95,137,901	8275	2.9 (0.1)
6	Tea	2,584,194	293	2.6 (0.3)	Tea	9,519,279	1070	2.5 (0.2)	Soft drinks	92,330,988	8918	2.8 (0.1)
7	Cheese	2,261,411	265	2.3 (0.2)	Diet soft drinks	8,358,603	685	2.2 (0.1)	Tea	73,796,069	6327	2.3 (0.1)
8	Sugar substitutes	2,220,376	326	2.2 (0.3)	Soft drinks	7,651,814	957	2.0 (0.1)	Other vegetables and combinations	61,835,369	5004	1.9 (0.0)
9	Eggs and omelets	2,000,524	255	2.0 (0.2)	Other vegetables and combinations	7,539,753	823	2.0 (0.1)	Sugars and honey	60,595,579	6302	1.9 (0.1)
10	Cold cuts and cured meats	1,973,622	222	2.0 (0.2)	Cream and cream substitutes	7,361,081	858	2.0 (0.1)	Lettuce and lettuce salads	60,358,697	4650	1.9 (0.0)

^1^ The What We Eat in America Food Categories were applied to categorize all foods and beverages to 151 subcategories; ^2^ The ranked list of the top 10 most frequently reported food subcategories for the group reporting taking insulin, those with T2D but not using insulin, and participants without diabetes were not statistically different (*p*-value = 0.73 as indicated by the Kruskal–Wallis test); ^3^ The sum of the estimated weighted frequencies for all type foods or beverages reported in 1 day among adult participants ≥18 years of NHANES 2009–2016; ^4^ Survey weights and adjustments for the complex survey design were applied to represent the non-institutionalized U.S. population; ^5^ The frequency that a food or beverage was reported without sample weights; ^6^ Derived from the weighted frequency of the food or beverage subcategories divided by the total weighted frequency of all foods and beverages subcategories (*n*) reported by whole sample in a single day, where *n* = 98,959,094 for the group reporting taking insulin, *n* = 376,469,886 for the group reporting T2D w/o taking insulin, and *n* = 3,245,557,852 for the group w/o diabetes. Estimated weighted percentage has been abbreviated to “Wtd %”; SE stands for standard error.

**Table 4:** Top 10 most frequently consumed What We Eat In America food or beverage categories ^1^, unweighted and weighted frequency of reported food or beverage categories, percentage of the reported foods or beverage category out of total, and standard error of the percentage of reported foods or beverage represented by the category among all reported foods or beverage categories for U.S. adults 18 years or older taking insulin, with type 2 diabetes but not taking insulin, and without diabetes drawn from 2009–2016 NHANES data ^2^.

	The Group Reporting Taking Insulin (*n* = 774)				The Group Reporting T2D w/o Insulin (*n* = 2758)				The Group w/o Diabetes (*n* = 17,796)			
Rank	What We Eat in America Category ^1^	Weighted Frequency ^3,4^	Frequency ^5^	Wtd% ^4,6^ (SE)	What We Eat in America Category ^1^	Weighted Frequency ^3,4^	Frequency ^5^	Wtd% ^4,6^ (SE)	What We Eat in America Category ^1^	Weighted Frequency ^3,4^	Frequency ^5^	Wtd% ^4,6^ (SE)
Total		98,578,506	12,039			374,707,908	43,155			3,232,070,662	275,796	
1	Plain water	9,697,772	1185	9.8 (0.4)	Plain water	36,973,717	4429	9.9 (0.3)	Plain water	319,959,647	27,493	9.9 (0.2)
2	Vegetables w/o white potato	9,059,302	982	9.2 (0.6)	Vegetables w/o white potato	33,386,898	3683	8.9 (0.3)	Vegetables w/o white potato	274,695,816	22,064	8.5 (0.1)
3	Coffee & tea	6,993,415	853	7.1 (0.3)	Coffee & tea	26,580,326	3167	7.1 (0.2)	Coffee & tea	210,481,073	17,554	6.5 (0.1)
4	Fats & oil	6,975,164	799	7.1 (0.4)	Fats & oils	24,735,148	2599	6.6 (0.2)	Fats & oils	196,833,742	15,227	6.1 (0.1)
5	Breads, rolls, tortillas	5,814,047	704	5.9 (0.3)	Breads, rolls, tortillas	21,868,818	2632	5.8 (0.1)	Breads, rolls, tortillas	161,270,439	14,243	5.0 (0.1)
6	Fruits	4,539,100	604	4.6 (0.3)	Fruits	17,789,900	2199	4.7 (0.2)	Fruits	150,617,266	12,803	4.7 (0.1)
7	Diet beverages	4,191,552	449	4.3 (0.4)	Condiments and sauces	15,875,817	1788	4.2 (0.2)	Condiments and sauces	148,905,014	12,777	4.6 (0.1)
8	Condiments and sauces	4,117,112	470	4.2 (0.3)	Sugars	14,422,141	1877	3.8 (0.2)	Sweetened beverages	144,772,013	14,108	4.5 (0.1)
9	Sugar	3,757,238	562	3.8 (0.3)	Milk	13,242,566	1459	3.5 (0.2)	Sugars	110,810,638	10,222	3.4 (0.1)
10	Cured meats/poultry	3,366,292	397	3.4 (0.3)	Sweet bakery product	12,011,790	1339	3.2 (0.1)	Milk	103,668,977	8770	3.2 (0.1)

^1^ The What We Eat in America Food Categories were applied to categorize all foods and beverages to 46 categories; ^2^ The ranked list of the top 10 most frequently reported food categories for the group reporting taking insulin, those with T2D but not using insulin, and participants without diabetes were not statistically different (*p*-value = 0.96 as indicated by the Kruskal–Wallis test); ^3^ The sum of the estimated weighted frequencies for all type foods or beverages reported in 1 day among adult participants ≥18 years of NHANES 2009–2016; ^4^ Survey weights and adjustments for the complex survey design were applied to represent 1 non-institutionalized U.S. population; ^5^ The frequency that a food or beverage was reported without sample weights; ^6^ Derived from the weighted frequency of the food or beverage categories divided by the total weighted frequency of all foods and beverages categories (*n*) reported by whole sample in a single day, where *n* = 98,578,506 for the group reporting taking insulin, *n* = 374,707,908 for the group reporting T2D w/o taking insulin, and *n* = 3,232,070,662 for the group w/o diabetes. Estimated weighted percentage has been abbreviated to “Wtd %”; SE stands for standard error.

**Table 5:** Top 10 highest energy contributing food items or beverages ^1^, the unweighted frequency and weighted energy contribution of reported food items or beverages, percentage of the total represented by the reported food or beverage items ^1^, and standard error of the percentage of the total represented by the reported food or beverage items among all reported food or beverage items for U.S. adults 18 year or older taking insulin, with type 2 diabetes but not taking insulin, and without diabetes drawn from 2009–2016 NHANES data ^2^.

	The Group Reporting Taking Insulin (*n* = 774)				The Group Reporting T2D w/o Insulin (*n* = 2758)				The Group w/o Diabetes (*n* = 17,796)			
Rank	What We Eat in America item ^1^	Weighted Energy Contribution ^3,4^	Frequency ^5^	Wtd% ^4,6^ (SE)	What We Eat in America item ^1^	Weighted Energy Contribution ^3,4^	Frequency ^5^	Wtd% ^4,6^ (SE)	What We Eat in America item ^1^	Weighted Energy Contribution ^3,4^	Frequency ^5^	Wtd% ^4,6^ (SE)
Total		11,173,326,140	10,655			45,994,398,876	38,344			438,552,576,789	246,981	
1	2% reduced fat milk	175,158,078	187	1.5 (0.3)	Soft drink, cola-type	620,925,847	460	1.4 (0.1)	Soft drink, cola-type	7,727,219,654	4172	1.8 (0.1)
2	Wheat or cracked wheat bread	107,505,626	85	1.0 (0.6)	2% reduced fat milk	542,124,845	613	1.2 (0.1)	Beer (include ale)	7,624,446,189	1643	1.7 (0.1)
3	Soft white roll	100,546,094	54	0.9 (0.2)	Whole milk	495,105,225	345	1.1 (0.2)	French fries	4,536,464,186	1608	1.0 (0.0)
4	Regular ice cream (not chocolate)	97,758,809	28	0.9 (0.3)	Beer (include ale)	446,611,096	133	1.0 (0.2)	2% reduced fat milk	4,370,770,940	3298	1.0 (0.1)
5	White bread	92,313,363	100	0.8 (0.1)	French fries	390,088,888	171	0.8 (0.1)	Beer, lite	4,119,958,979	1005	0.9 (0.1)
6	Whole wheat bread	88,551,645	59	0.8 (0.2)	Banana	370,150,703	530	0.8 (0.1)	Ice cream (not chocolate)	3,839,785,911	1133	0.9 (0.1)
7	Soft drink, cola-type	85,779,001	77	0.8 (0.2)	Ice cream (not chocolate)	358,175,595	157	0.8 (0.1)	Banana	3,167,442,419	2737	0.7 (0.0)
8	White potato	79,297,997	56	0.7 (0.1)	White bread	339,050,117	328	0.7 (0.1)	Whole milk	3,063,855,299	2338	0.7 (0.0)
9	Banana	77,782,509	125	0.7 (0.1)	Soft white roll	293,239,277	242	0.6 (0.1)	White bread	3,028,693,277	1902	0.7 (0.0)
10	Whole milk	77,426,678	85	0.7 (0.1)	Wheat or cracked wheat bread	267,359,710	275	0.6 (0.1)	Soft drink, fruit-flavored, caffeine free	2,649,165,951	2007	0.6 (0.0)

^1^ 6772 food or beverage items were reported by participants from NHANES 2009–2016; ^2^ The ranked list of the top 10 highest energy contributing food items for the participants reporting taking insulin, those with T2D but not using insulin, and participants without diabetes were not statistically different (*p*-value = 0.11 as indicated by the Kruskal–Wallis test); ^3^ The sum of the estimated weighted energy contribution for all type foods or beverages reported in 1 day among adult participants ≥18 years of NHANES 2009–2016; ^4^ Survey weights and adjustments for the complex survey design were applied to represent the non-institutionalized U.S. population; ^5^ The frequency that a food or beverage was reported without sample weights; ^6^ Derived from the weighted energy contribution of the individual food or beverage divided by the total weighted energy contribution of all foods and beverages (*n*) reported by whole sample in a single day, where *n* = 11,173,326,140 for the group reporting taking insulin, *n* = 45,994,398,876 for the group reporting T2D w/o taking insulin, and *n* = 438,552,576,789 for the group w/o diabetes. Estimated weighted percentage has been abbreviated to “Wtd %”; SE stands for standard error.

**Table 6:** Top 10 highest energy contributing What We Eat in America food and beverage subcategories ^1^, the unweighted frequency and weighted energy contribution of reported foods items or beverages, percentage of the total represented by the reported food or beverage subcategory, and standard error of the percentage of the total represented by the reported food or beverage subcategories among all reported food or beverage subcategories for U.S. adults 18 year or older taking insulin, with type 2 diabetes but not taking insulin, and without diabetes drawn from 2009–2016 NHANES data ^2^.

	The Group Reporting Taking Insulin (*n* = 774)				The Group Reporting T2D w/o Insulin (*n* = 2758)				The Group w/o Diabetes (*n* = 17,796)			
Rank	What we Eat in America subcategory ^1^	Weighted Energy Contribution ^3,4^	Frequency ^5^	Wtd% ^4,6^ (SE)	What we Eat in America subcategory ^1^	Weighted Energy Contribution ^3,4^	Frequency ^5^	Wtd% ^4,6^ (SE)	What we Eat in America subcategory ^1^	Weighted Energy Contribution ^3,4^	Frequency ^5^	Wtd% ^4,6^ (SE)
Total		11,173,326,140	10,655			45,994,398,876	38,344			438,552,576,789	246,981	
1	Yeast breads	542,059,395	449	4.9 (0.3)	Yeast breads	2,015,776,561	1529	4.4 (0.2)	Pizza	17,155,217,138	2325	3.9 (0.2)
2	Pizza	352,519,439	74	3.2 (0.6)	Pizza	1,630,091,499	249	3.5 (0.5)	Soft drinks	16,878,162,486	8918	3.8 (0.1)
3	Chicken, whole pieces	344,627,978	182	3.1 (0.5)	Cakes and pies	1,406,511,677	386	3.1 (0.3)	Yeast breads	15,025,292,371	8275	3.4 (0.1)
4	Eggs and omelets	322,535,347	255	2.9 (0.3)	Chicken, whole pieces	1,276,775,627	731	2.8 (0.2)	Beer	12,606,044,121	2790	2.9 (0.1)
5	Meat mixed dishes	279,742,218	105	2.5 (0.4)	Soft drinks	1,266,355,047	957	2.8 (0.2)	Chicken, whole pieces	11,830,893,925	4719	2.7 (0.1)
6	Cakes and pies	258,928,280	91	2.3 (0.4)	Cookies and brownies	1,132,693,357	630	2.5 (0.2)	Nuts and seeds	11,385,283,950	3925	2.6 (0.1)
7	Cookies and brownies	245,466,831	185	2.2 (0.2)	Nuts and seeds	1,129,967,899	563	2.5 (0.2)	Burritos and tacos	10,936,204,042	1480	2.5 (0.1)
8	Cheese	241,035,357	265	2.2 (0.3)	Burritos and tacos	1,116,149,548	203	2.4 (0.4)	Cookies and brownies	10,449,344,792	4386	2.4 (0.1)
9	Burritos and tacos	238,780,285	49	2.1 (0.5)	Eggs and omelets	1,062,625,029	770	2.3 (0.1)	Cakes and pies	10,399,896,397	2373	2.4 (0.1)
10	Cold cuts and cured meats	218,354,393	222	2.0 (0.4)	Meat mixed dishes	980,422,000	371	2.1 (0.2)	Cheese	9,908,844,842	7342	2.3 (0.1)

^1^ The What We Eat in America Food Categories were applied to categorize all foods and beverages to 151 subcategories; ^2^ The ranked list of the top 10 highest energy contributing food subcategories for the participants reporting taking insulin, those with T2D but not using insulin, and participants without diabetes were not statistically different (*p*-value = 0.34 as indicated by the Kruskal–Wallis test); ^3^ The sum of the estimated weighted energy contribution for all types of foods or beverages reported in 1 day among adult participants ≥18 years of NHANES 2009–2016; ^4^ Survey weights and adjustments for the complex survey design were applied to represent the non-institutionalized U.S. population; ^5^ The frequency that a food or beverage was reported without sample weights; ^6^ Derived from the weighted energy contribution of the food or beverage subcategories divided by the total weighted energy contribution of all foods and beverages subcategories (*n*) reported by whole sample in a single day, where *n* = 11,173,326,140 for the group reporting taking insulin, *n* = 45,994,398,876 for the group reporting T2D w/o taking insulin, and *n* = 438,552,576,789 for the group w/o diabetes. Estimated weighted percentage has been abbreviated to “Wtd %”; SE stands for standard error.

**Table 7:** Top 10 highest energy contributing What We Eat in America food and beverage categories ^1^, the unweighted frequency and weighted energy contribution of reported food items or beverages, percentage of the total represented by the reported food or beverage category, and standard error of the percentage of the total represented by the reported food or beverage categories among all reported food or beverage categories for U.S. adults 18 year or older taking insulin, with type 2 diabetes but not taking insulin, and without diabetes drawn from 2009–2016 NHANES data ^2^.

	The Group Reporting Taking Insulin (*n* = 774)				The Group Reporting T2D w/o Insulin (*n* = 2758)				The Group w/o Diabetes (*n* = 17,796)			
Rank	What We Eat in America category ^1^	Weighted Energy Contribution ^3,4^	Frequency ^5^	Wtd% ^4,6^ (SE)	What We Eat in America category ^1^	Weighted Energy Contribution ^3,4^	Frequency ^5^	Wtd% ^4,6^ (SE)	What We Eat in America category ^1^	Weighted Energy Contribution ^3,4^	Frequency ^5^	Wtd% ^4,6^ (SE)
Total		11,067,583,100	10,599			45,507,634,682	38,078			434,779,439,791	245,533	
1	Breads, rolls, tortillas	923,994,769	704	8.3 (0.4)	Breads, rolls, tortillas	3,653,085,399	2632	8.0 (0.2)	Sweet bakery product	27,904,006,131	9010	6.4 (0.1)
2	Sweet bakery product	629,785,636	341	5.7 (0.5)	Sweet bakery product	3,257,752,964	1339	7.2 (0.4)	Breads, rolls, tortillas	27,497,056,987	14,243	6.3 (0.1)
3	Mixed dishes- sandwiches	550,338,991	155	5.0 (0.6)	Sweetened beverages	2,041,686,370	1518	4.5 (0.2)	Sweetened beverages	26,867,482,295	14,106	6.2 (0.1)
4	Mixed dishes-meat, poultry, seafood	487,328,523	194	4.4 (0.5)	Mixed dishes- sandwiches	1,918,027,605	477	4.2 (0.3)	Alcoholic beverages	21,928,960,736	5463	5.0 (0.2)
5	Fats & oils	449,849,893	799	4.1 (0.3)	Mixed dishes- meat, poultry, seafood	1,846,139,541	703	4.1 (0.3)	Mixed dishes- sandwiches	18,966,260,477	3297	4.4 (0.1)
6	Poultry	441,972,521	233	4.0 (0.5)	White potatoes	1,762,408,554	842	3.9 (0.2)	Mixed dishes- pizza	17,155,217,138	2325	3.9 (0.2)
7	Cured meats/poultry	427,895,678	397	3.9 (0.4)	Plant based protein foods	1,711,721,004	1041	3.8 (0.2)	Mixed dishes- Mexican	16,503,304,178	2545	3.8 (0.2)
8	White potatoes	377,782,656	216	3.4 (0.4)	Poultry	1,654,562,156	915	3.6 (0.2)	Poultry	16,063,883,380	6066	3.7 (0.1)
9	Mixed dishes- Mexican	362,937,339	96	3.3 (0.6)	Mixed dishes- pizza	1,630,091,499	249	3.6 (0.5)	Plant based protein foods	15,960,314,784	6477	3.7 (0.1)
10	Sweetened beverages	361,977,279	336	3.3 (0.3)	Mixed dishes- Mexican	1,602,969,844	361	3.5 (0.4)	Mixed dishes- grain based	15,334,421,326	2991	3.5 (0.1)

^1^ The What We Eat in America Food Categories were applied to categorize all foods and beverages to 46 categories; ^2^ The ranked list of the top 10 highest energy contributing food categories for the participants reporting taking insulin, those with T2D but not using insulin, and participants without diabetes were not statistically different (*p*-value = 0.61 as indicated by the Kruskal–Wallis test); ^3^ The sum of the estimated weighted energy contribution for all type foods or beverages categories reported in 1 day among adult participants ≥18 years of NHANES 2009–2016; ^4^ Survey weights and adjustments for the complex survey design were applied to represent the non-institutionalized U.S. population; ^5^ The frequency that a food or beverage was reported without sample weights; ^6^ Derived from the weighted energy contribution of the food or beverage category divided by the total weighted energy contribution of all foods and beverage categories (*n*) reported by whole sample in a single day, where *n* = 11,067,583,100 for the group reporting taking insulin, *n* = 45,507,634,682 for the group reporting T2D w/o taking insulin, and *n* = 434,779,439,791 for the group w/o diabetes. Estimated weighted percentage has been abbreviated to “Wtd %”; SE stands for standard error.

**Table 8:** Broad What We Eat In America food and beverage category ^1^ intake comparisons by frequency and energy among U.S. adults 18 year or older taking insulin, with type 2 diabetes but not taking insulin, and without diabetes drawn from NHANES 2009–2016 ^2^.

	The Group Reporting Taking Insulin (*n* = 774)		The Group Reporting T2D w/o Insulin (*n* = 2758)		the Group w/o Diabetes (*n* = 17,796)		*X*^2^ *p*-Value	
Broad WWEIA food category ^1^	Wtd % ^3^ of reported foods (SE)	Wtd % ^3^ of reported energy (SE)	Wtd % ^3^ of reported foods (SE)	Wtd % ^3^ of reported energy (SE)	Wtd % ^3^ of reported foods (SE)	Wtd % ^3^ of reported energy (SE)	Frequency ^4^	Energy ^4^
Milk/Dairy ^5^	6.9 (0.4)	6.9 (0.5)	7.1 (0.2)	6.6 (0.3)	7.4 (0.1)	6.3 (0.1)	0.1	0.3
Protein ^6^	11.4 (0.5)	18.8 (1.0)	11.2 (0.2)	17.2 (0.4)	10.6 (0.1)	15.7 (0.2)	0.01	<0.0001 *
Mixed Dish ^7^	6.9 (0.4)	22.2 (1.2)	6.9 (0.2)	21.7 (0.6)	7.5 (0.1)	22.7 (0.3)	0.004	0.3
Grain ^8^	9.7 (0.3)	15.2 (0.6)	9.6 (0.2)	14.3 (0.4)	8.8 (0.1)	12.1 (0.2)	<0.0001 *	<0.0001 *
Snack/Sweet ^9^	8.5 (0.4)	13.8 (0.8)	9.5 (0.2)	15.4 (0.3)	10.2 (0.1)	15.1 (0.2)	<0.0001 *	0.2
Fruit ^10^	4.6 (0.3)	2.8 (0.2)	4.7 (0.2)	2.7 (0.1)	4.6 (0.1)	2.5 (0.1)	0.9	0.05
Vegetable ^11^	11.0 (0.6)	6.1 (0.5)	11.0 (0.3)	6.3 (0.2)	10.4 (0.1)	5.4 (0.1)	0.1	0.0009 *
Beverage ^12^	14.8 (0.3)	6.1 (0.4)	13.9 (0.3)	7.3 (0.3)	13.8 (0.1)	9.5 (0.2)	0.09	<0.0001 *
Alcohol ^13^	0.9 (0.2)	2.4 (0.5)	1.2 (0.1)	2.9 (0.3)	2.1 (0.1)	5.0 (0.2)	<0.0001 *	<0.0001 *
Water ^14^	10.2 (0.4)	0.0 (0.0)	10.1 (0.4)	0.0 (0.0)	10.2 (0.2)	0.1 (0.0)	0.9	0.0011 *
Fat/Oil ^15^	7.1 (0.4)	4.0 (0.3)	6.6 (0.2)	3.5 (0.1)	6.1 (0.1)	3.2 (0.1)	0.002	0.003
Cond ^16/^ Sauce ^17^	4.2 (0.3)	0.9 (0.1)	4.2 (0.2)	0.9 (0.1)	4.6 (0.1)	1.0 (0.0)	0.09	0.6
Sugars ^18^	3.8 (0.3)	0.8 (0.1)	3.8 (0.2)	1.1 (0.1)	3.4 (0.1)	1.2 (0.0)	0.05	0.08
Other	0.2 (0.1)	0.1 (0.0)	0.2 (0.0)	0.1 (0.0)	0.4 (0.0)	0.3 (0.0)	0.006	<0.0001 *

^1^ The What We Eat in America broad food categories were applied to categorize all foods and beverages reported in a single day to 14 broad food categories; ^2^ Survey weights and adjustments for the complex survey design were applied to represent the non-institutionalized U.S. population. Total numbers and percentages do not always add up to sample size due to missing values and rounding; ^3^ Wtd % stands for the estimated weighted percentage of all reports of foods or beverages or energy from reported foods or beverages reported in a single day that are included in a food category; ^4^ *p*-values were calculated using the Rao–Scott modified chi-square statistic; * indicates *p* < 0.05/42 or *p* < 0.0012 using a Bonferroni type adjustment for multiple comparisons for broad food category intake among 14 broad food categories × 3 groups; ^5^ Milk, flavored milk, dairy drinks and substitutes, cheese and yogurt; ^6^ Meats, poultry, seafood, eggs, cured meats/poultry, and plant-based protein foods; ^7^ Mixed dishes containing meat, poultry seafood; grain-based; Asian; Mexican; pizza; sandwiches, and soups; ^8^ Cooked grains, breads, rolls, tortillas, quick breads and bread products, ready-to-eat cereals, and cooked cereals; ^9^ Savory snacks, crackers, snack/meal bars, sweet bakery products, candy and other desserts; ^10^ Fresh fruits, dried fruits, and fruit salads; ^11^ Vegetables and white potatoes; ^12^ 100% juice, diet beverages, sweetened beverages, coffee and tea; ^13^ Beer, wine, liquor and cocktails; ^14^ Plain water and flavored or enhanced water; ^15^ Butter and animal fats, margarine, cream cheeses, cream, mayonnaise, salad dressings and vegetable oils; ^16^ Condiment; ^17^ Tomato-based, soy-based, mustard, olives, pickled vegetables, pasta sauces, dips, gravies, and other sauces; ^18^ Sugars, honey, jams, syrups, and toppings.

## Data Availability

Data described in the manuscript are publicly and freely available without restriction at https://www.cdc.gov/nchs/nhanes/index.htm (accessed on 14 July 2021). Analytic code is available upon request.
